# SARS-CoV-2 neutralizing antibody response after three doses of mRNA1273 vaccine and COVID-19 in hemodialysis patients

**DOI:** 10.3389/fneph.2022.926635

**Published:** 2022-07-22

**Authors:** Xiaoling Wang, Maggie Han, Lemuel Rivera Fuentes, Ohnmar Thwin, Nadja Grobe, Kevin Wang, Yuedong Wang, Peter Kotanko

**Affiliations:** ^1^ Renal Research Institute, New York, NY, United States; ^2^ College of Creative Studies, University of California at Santa Barbara, Santa Barbara, CA, United States; ^3^ Department of Statistics and Applied Probability, University of California at Santa Barbara, Santa Barbara, CA, United States; ^4^ Icahn School of Medicine at Mount Sinai, New York, NY, United States

**Keywords:** coronavirus, COVID-19, hemodialysis, mRNA1273, neutralizing antibody, SARS-CoV-2, vaccination, Omicron

## Abstract

**Background:**

In hemodialysis patients, a third vaccination is frequently administered to augment protection against coronavirus disease 2019 (COVID-19). However, the newly emerged B.1.1.159 (Omicron) variant may evade vaccinal protection more easily than previous strains. It is of clinical interest to better understand the neutralizing activity against severe acute respiratory syndrome coronavirus 2 (SARS-CoV-2) variants after booster vaccine or COVID-19 infection in these mostly immunocompromised patients.

**Methods:**

Hemodialysis patients from four dialysis centers were recruited between June 2021 and February 2022. Each patient provided a median of six serum samples. SARS-CoV-2 neutralizing antibodies (nAbs) against wild type (WT) or Omicron were measured using the GenScript SARS-CoV-2 Surrogate Virus Neutralization Test Kit.

**Results:**

Forty-two patients had three doses of mRNA1273. Compared to levels prior to the third dose, nAb-WT increased 18-fold (peak at day 23) and nAb-Omicron increased 23-fold (peak at day 24) after the third dose. Peak nAb-WT exceeded peak nAb-Omicron 27-fold. Twenty-one patients had COVID-19 between December 24, 2021, and February 2, 2022. Following COVID-19, nAb-WT and nAb-Omicron increased 12- and 40-fold, respectively. While levels of vaccinal and post-COVID nAb-WT were comparable, post-COVID nAb-Omicron levels were 3.2 higher than the respective peak vaccinal nAb-Omicron. Four immunocompromised patients having reasons other than end-stage kidney disease have very low to no nAb after the third dose or COVID-19.

**Conclusions:**

Our results suggest that most hemodialysis patients have a strong humoral response to the third dose of vaccination and an even stronger post-COVID-19 humoral response. Nevertheless, nAb levels clearly decay over time. These findings may inform ongoing discussions regarding a fourth vaccination in hemodialysis patients.

## Introduction

In the general population, severe acute respiratory syndrome coronavirus 2 (SARS-CoV-2) antibody titers decay over time after two vaccine doses or SARS-CoV-2 infection (1). Dialysis patients and kidney transplant recipients are at an even greater risk for a lower antibody level and pronounced decline for vaccinal SARS-CoV-2 antibodies (2–4). Therefore, a third vaccination (“booster”) is frequently administered to augment protection against coronavirus disease 2019 (COVID-19) in these patients. Previous reports described receptor-binding domain (RBD) immunoglobulin G (IgG) and neutralizing antibodies (nAbs) after three doses of vaccinations with AZD1222 (AZD; Oxford–AstraZeneca), BNT162b2 (BNT; Pfizer–BioNTech), or mRNA1273 (MOD; Moderna) following various regimens, such as BNT-BNT-MOD, BNT-BNT-BNT, and AZT-BNT-BNT in these dialysis patients (5–9). While MOD is widely used in hemodialysis patients, we are unaware of data on the nAb response to MOD-MOD-MOD. Also, the newly emerged B.1.1.159 (Omicron) variant harbors more than 30 mutations in its spike protein and may thus evade vaccinal protection more easily than previous strains. Omicron-BA.1 has been the predominant variant at New York City area since middle of December 2021. Local genomic sequencing data indicated that it accounted for 91% of COVID-19 cases on December 25, 2021; the rate was 99% on January 8, 2022, and remained above 98% throughout January 2022 (10). In the general population, vaccinal antibodies show lower neutralizing activities against Omicron than other SARS-CoV-2 strains (11). The decline in Omicron neutralization is similar in hemodialysis patients, albeit data are limited (12). It is of clinical interest to better understand the neutralizing activity of vaccinal and post-COVID-19 nAbs against SARS-CoV-2 variants in these mostly immunocompromised patients. To that end, we set up a test system to quantitate nAbs against both SARS-CoV-2 wild type (WT) and Omicron in the same blood sample.

## Materials and methods

### Participants

Between June 2021 and February 2022, 236 in-center hemodialysis patients from four dialysis centers in Manhattan, New York City, were recruited for this institutional review board (IRB)-approved study (Western Institutional Research Board, numbers 1311160 and 1305892). Patients underwent nasopharyngeal or oral swab SARS-CoV-2 RT-PCR testing if they had fever or other signs and symptoms indicative of COVID-19, returned from domestic or international travel, had recent exposure to a COVID-19 patient, returned from a hospitalization, or had a positive home antigen test.

### Neutralizing antibody measurement

SARS-CoV-2 surrogate nAbs were measured with the GenScript SARS-CoV-2 Surrogate Virus Neutralization Test Kit (Cat#L00847-A). For nAbs against WT SARS-CoV-2 virus (nAb-WT), horseradish peroxidase (HRP)-conjugated WT RBD of SARS-CoV-2 spike protein (WT-RBD-HRP, GenScript Cat#Z03594, constructed from Arg319 to Ser591 of WT spike protein) was used. Series dilution of WT IgG antibody standard (GenScript Cat#A02087) from 600 to 9.375 U/ml was performed simultaneously to calculate each sample’s nAb-WT amount. Inhibition concentration at 50% (IC50) of WT IgG antibody standard was determined by 10 individual experiments. The surrogate virus neutralization titer at 50% (sVNT50) was calculated as nAb/IC50_standard_. sVNT50-Omicron was assessed similarly, except for the use of Omicron-RBD-HRP (GenScript Cat#Z03730, constructed from Arg319 to Phe541 of Omicron spike protein) and Omicron IgG antibody standard (GenScript Cat# A02161, series dilution from 2,400 to 37.5 U/ml). To compare sVNT50-WT and sVNT50-Omicron, we measured the WT-RBD-HRP and Omicron-RBD-HRP protein concentrations using absorbance at 280 nm (Nanodrop One, Thermo Fisher) and normalized them to the same level.

### Neutralizing antibody data analysis

#### Vaccinal neutralizing antibody analysis

We fit a semiparametric mixed-effects model with a change point at time zero (i.e., the day of vaccination), an adaptive spline, and a random intercept for each subject to sVNT50 on a log_10_ scale and estimated the time-to-peak and its 95% confidence interval using bootstrapping (13). We fit a linear mixed-effects model to estimate post-peak sVNT50 monoexponential decay rates and half-lives.

#### Post-COVID-19 neutralizing antibody analysis

We illustrate the temporal evolution of post-COVID-19 sVNT50 in two complementary ways:

a) Time series analysis Twenty-one patients provided 82 serum samples between 60 days before and 40 days after COVID-19 diagnosis. Each patient provided a median of three samples (range 1–11).

b) Box-and-whisker plots and titer levels. For these figures and the titer calculation, we consider sera collected between 45 days before and 30 days after COVID-19 diagnosis. Most patients provide multiple samples during that time. We selected samples that were closest before the COVID-19 diagnosis date and the latest sample within the 30-day post-diagnosis window. Twenty patients provided 20 pre- and 17 post-COVID-19 samples. In three patients, no post-COVID-19 samples were available.

## Results

**Figure 1:**
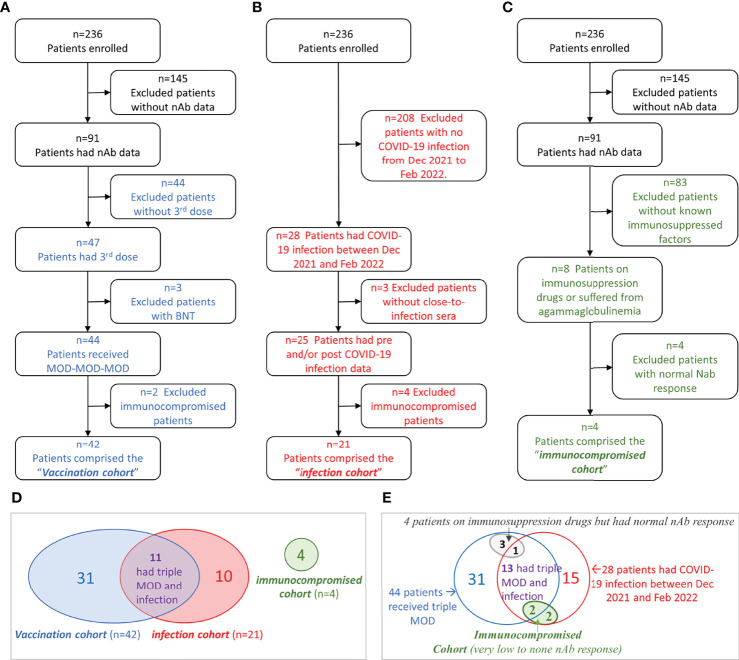
Flowchart of cohort selection and Venn diagrams of the patient groups. **(A–C)** Flowchart of patient selection of the vaccination cohort **(A)**, infection cohort **(B)**, and immunocompromised cohort **(C)**. **(D)** Venn diagram showing the patient overlap between the three cohorts. **(E)** Venn diagram showing the relationships among immunocompromised status (other than kidney disease), vaccination, and COVID-19 infection in hemodialysis (HD) patients.

Between June 2021 and February 2022, we measured nAb titers in 421 serial serum samples of 91 chronic hemodialysis patients from four dialysis centers located in Manhattan, New York City. The selection process of each study cohort was illustrated in **Figures 1A–C**. In summary, 42 patients who received MOD-MOD-MOD comprised the “vaccination cohort.” Twenty-one patients who had COVID-19 between December 24, 2021, and February 1, 2022, comprised the “infection cohort.” Four severely immunocompromised patients having very low to no sVNT50 after the third dose or COVID-19 due to reasons other than end-stage kidney disease comprised the “immunocompromised cohort.” Adding together, a total of 332 serial serum samples from 56 patients (median six samples per patient, range 2–15) were used for the entire analysis (**Figure 1D**). Thirteen patients had COVID-19 breakthrough infection after receiving MOD-MOD-MOD, including two patients in the immunocompromised cohort (**Figure 1E**).

### Vaccinal neutralizing antibody analysis

**Figure 2:**
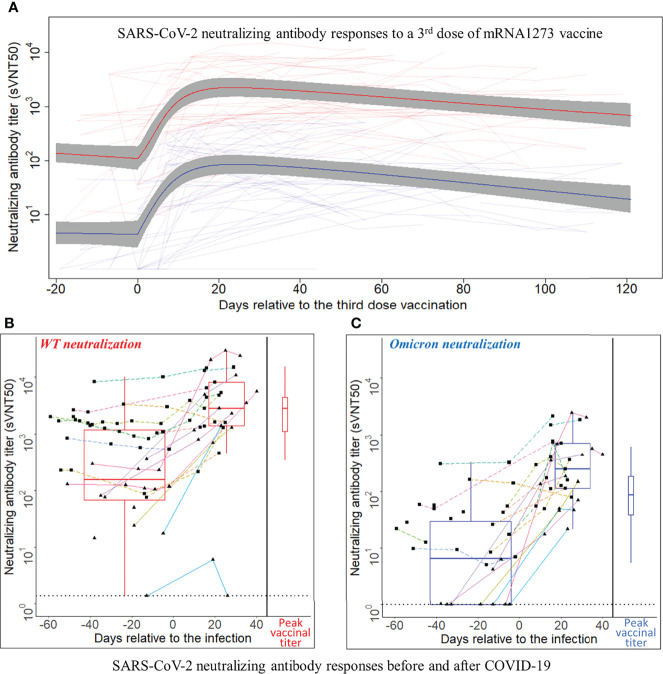
SARS-CoV-2 neutralizing antibody responses to a third dose of mRNA1273 vaccine or COVID-19 infection. **(A)** SARS-CoV-2 neutralizing antibody responses to a third dose of mRNA1273 vaccine. Day 0 is the third dose administration date. The Y-axis is plotted on a log_10_ scale. Thin light red and light blue lines connect individual patient-level sVNT50 against wild type (WT) and Omicron, respectively. The heavy lines are estimates of population mean trajectories gleaned from the semiparametric mixed-effects model for sVNT50-WT (red) and sVNT50-Omicron (blue) time series data, respectively. The gray area represents the 95% confidence intervals. **(B, C)** SARS-CoV-2 neutralizing antibody responses before and after COVID-19. Panel **(B)** shows the sVNT50-WT, and panel **C** shows the sVNT50-Omicron. Day 0 is the date of COVID-19 diagnosis. The Y-axis is plotted on a log_10_ scale. For the time series depiction, each patient contributed between one and 11 samples in a time interval from 60 days pre- to 40 days after COVID-19 diagnosis. The gray dotted line indicates the assay detection limits. Measurements from the same patient are connected by a straight line. Each color indicates an individual patient. Solid lines with triangle dots indicate patients without a third vaccine dose. Dashed lines with square dots indicate patients who received a third vaccine dose. The box-and-whisker plots build on titers closest before COVID-19 diagnosis and latest in the first post-diagnosis month. For comparison, the peak titers after the third vaccine dose (red for sVNT50-WT, blue for sVNT50-Omicron) are drawn at the right side of each panel as box-and-whisker plots.

Time series of sVNT50-WT and sVNT50-Omicron were plotted and fitted using semiparametric mixed-effects models (**Figure 2A**). To ensure that the models were built on using uninfected patients after the third vaccine dose administration, 19 data points from nine PCR-confirmed COVID-19 patients were excluded after the day of COVID-19 diagnosis. In addition, we identified 10 samples in seven patients with suspected asymptomatic COVID-19 by applying a heuristic that flagged samples with an sVNT50 increased by more than 30% as compared to the preceding level at least 2 months after the third dose. These 10 points were removed as well. The final vaccinal cohort comprised 42 patients contributing 271 samples. Before receiving their third MOD dose, all patients showed detectable sVNT50-WT (geometric mean 126); however, 16 patients had sVNT50-Omicron below the detection limit (geometric mean 4). After the third MOD dose, all but one patient showed detectable sVNT50 against SARS-CoV-2 WT and Omicron; this patient had detectable sVNT50-WT only. Following the third MOD dose, sVNT50-WT increased compared to the prior level 18-fold (peak at day 23) and sVNT50-Omicron increased 23-fold (peak at day 24). Peak sVNT50-WT exceeded peak sVNT50-Omicron 27-fold.

Twenty-six patients (62% of the vaccination cohort) received 0.5 ml MOD as their third dose, 16 (38%) received 0.25 ml, as per manufacturer’s recommendation issued on October 20, 2021 (https://www.fda.gov/media/153354/download). The small number of patients with either dose precluded a formal statistical analysis of vaccinal nAb kinetics; visual inspection of the nAb curves revealed no material differences (data not shown). Considering all 42 patients, half-lives of sVNT50-WT and sVNT50-Omicron were estimated to be 65 and 51 days, respectively (**Figure 2A**).

### Post-COVID-19 neutralizing antibody analysis

Eighty-two serum samples were available from the 21 patients in the infection cohort (median three per patient, range 1–11). Time series of sVNT50-WT and sVNT50-Omicron are shown in **Figures 2B, C**. Regarding sVNT50-WT, all patients except one had detectable sVNT50-WT pre-COVID-19; this nAb-negative patient was not vaccinated at the time of infection and developed only low-level nAb post-COVID-19 (**Figures 2B, C**, solid cyan lines bottom right). Regarding sVNT50-Omicron, seven patients had undetectable sVNT50-Omicron prior to COVID-19 (**Figure 2C**). All 21 patients had detectable sVNT50-Omicron post-COVID-19.

To better understand the nAb dynamics brought about by COVID-19, we investigated sVNT50-WT and sVNT50-Omicron in 37 serum samples collected between 45 days before and 30 days after COVID-19 diagnosis. These data are depicted in **Table 1** and as pre- and post-COVID-19 box-and-whisker plots in **Figures 2B, C** (for details, see *Materials and Methods*). Following COVID-19, sVNT50-WT and sVNT50-Omicron increased 12- and 40-fold, respectively. Vaccinal sVNT50-WT and post-COVID-19 sVNT50-WT were comparable; in contrast, post-COVID-19 sVNT50-Omicron was 3.2 higher than the respective peak vaccinal sVNT50-Omicron (**Table 1**; **Figures 2B, C**).

**Table 1:** Cohort baseline characteristics and neutralizing antibody responses to SARS-CoV-2 vaccination and COVID-19 infection.

		*Vaccination cohort*				*Infection cohort*	
Patients, n		42				21	
Age (years)		63 ± 10 (42–82)				62 ± 9 (41–80)	
Women, n (%)		11 (26%)				8 (38%)	
Race	African American, n (%)	32 (76%)				17 (81%)	
	White, n (%)	8 (19%)				3 (14%)	
	Asian, n (%)	2 (5%)				1 (5%)	
Vaccination Status		n = 26: MOD-MOD-MOD (0.5 ml) n = 16: MOD-MOD-MOD (0.25 ml)				3 unvaccinated; 2 BNT-BNT; 5 MOD-MOD; 11 MOD-MOD-MOD	
COVID Hospitalization, n (%)		Not appliable				3 (14%)	
COVID Mortality, n (%)		Not appliable				1 (5%)	
** **		**Titer Calculations**					
		**nAb titer** **before third MOD**	**Peak nAb titer** **after third MOD**	**Time-to-peak after third MOD (days)**	**nAb Half-life** **(days)**	**nAb titer** **pre-COVID-19** **(20 patients)**	**nAb titer** **post-COVID-19** **(17 patients)**
WT	Geometric mean	126	2,248	23	65	205	2,458
	95% CI	43–237	1,788–6,711	11–28	57–75	73–574	928–6,511
Omicron	Geometric mean	4	84	24	51	7	273
	95% CI	1–12	50–319	11–78	41–68	3–17	135–553

Neutralizing antibody (nAb) titer is presented as sVNT50 (surrogate virus neutralization titer at 50%). MOD, Moderna mRNA1273; BNT, Pfizer–BioNTech BNT162b2; CI, confidence interval. Age is shown as mean ± standard deviation.

Eleven out of 21 patients in the infection cohort had MOD-MOD-MOD prior to their infection (**Figures 2B, C**, dashed lines with square dots). The median days between the third dose and infection are 94 days (range 39–117). Prior to the infection, the sVNT50 of these 11 MOD-MOD-MOD patients was significantly higher than that of the remaining 10 patients (3 unvaccinated; 2 BNT-BNT; 5 MOD-MOD, solid lines with triangle dots in **Figures 2B, C**) (p < 0.001). After the infection, no significant sVNT50 difference was observed between the two groups.

### Neutralizing antibody in immunocompromised patients

Among 91 patients who had nAb data, eight patients either were on immunosuppressive drugs (N = 7) or suffered from agammaglobulinemia (N = 1). Their vaccinal and COVID-19 infection statuses were illustrated in the Venn diagram (**Figure 1E**). Among them, four patients (two tacrolimus, one infliximab, one mycophenolate mofetil and tacrolimus) had normal nAb response (**Figure 1E**, gray oval). The other four patients (one agammaglobulinemia, two hydroxychloroquine, one mycophenolate mofetil and tacrolimus) had very low to no nAb response (**Figure 1E**, green oval). These four patients contributed 14 serum samples that were excluded from the vaccinal and infection analyses described above. sVNT50-Omicron was negative in all 14 samples. The agammaglobulinemic patient developed very low vaccinal sVNT50-WT; the others, none. All four patients had COVID-19; their post-COVID-19 sVNT50-WT was either undetectable (N = 3) or very low (N = 1).

## Discussion

Our research in hemodialysis patients demonstrated a strong vaccinal nAb response to MOD-MOD-MOD and an even stronger one post-COVID-19. Although plaque reduction neutralization test (PRNT) is the preferred method for quantifying antibodies capable of neutralizing SARS-CoV-2 virus, multiple groups had extensively evaluated GenScript SARS-CoV-2 Surrogate Virus Neutralization Test Kit (6, 14–16). It was demonstrated that GenScript sVNT ELISA method gives comparable results and good correlation to PRNT in the detection of nAbs.

Our study has several strengths. First, while previous reports addressed the nAb response to various homologous and heterologous triple vaccination schemes (6–9, 12), this is the first regarding nAb dynamics after MOD-MOD-MOD vaccination. Second, we quantitated in the same serum sample the nAb responses specific to both WT and Omicron. Third, the study adds to our understanding of SARS-CoV-2 nAb in chronic hemodialysis patients by demonstrating that (a) after a third MOD dose, the vaccinal nAb response against Omicron is significantly less compared to the one against WT, an observation that may help us better understand breakthrough infections caused by Omicron; (b) while the time-to-peak is about the same for both nAb types, the half-life of nAb-Omicron tends to be shorter than that of nAb-WT; this finding may be related to a less durable nAb response against Omicron and inform ongoing discussions regarding the utility of a fourth vaccine dose in hemodialysis patients (17, 18). Our study also identified that 4 out of 91 hemodialysis patients (4.4%) had poor or absent immune responses. These patients may be suitable for an additional vaccine dose with a short interval and/or benefit from pre-exposure prophylaxis (2).

While SARS-CoV-2 genomic sequencing would have been desirable, concurrent local surveillance sequencing data make it likely that the Omicron BA.1 was the main cause of COVID-19 also in our patients (10). More Omicron variants such as BA.2, BA.3, BA.4, and BA.5 had emerged after February 2022. It would be interesting to see if a vaccine- or an infection-induced humoral response would be effective against the variants as well. As indicated earlier, the limited patient numbers prevented us from exploring relevant questions, for example, the impact of factors such as sex, race, and age on nAb kinetics. Also, larger patient cohorts are needed to probe into the relationship between nAb levels and COVID-19 severity. In summary, while hemodialysis patients are mostly considered immunocompromised, we found a strong vaccinal and—an even stronger—post-COVID-19 nAb response. Nevertheless, nAb levels clearly decay over time. If and to what extent nAb levels can inform personalized vaccination recommendations remains an open question that needs to be addressed in longitudinal studies that relate nAb levels to the risk of SARS-CoV-2 (re)infection.

## Data availability statement

The original contributions presented in the study are included in the article, further inquiries can be directed to the corresponding author.

## Ethics statement

The studies involving human participants were reviewed and approved by Western Institutional Research Board. The patients/participants provided their written informed consent to participate in this study.

## Funding

Research reported in this publication was supported by the National Institute of Diabetes and Digestive and Kidney Diseases of the National Institutes of Health, Award Number R01DK130067. The content is the sole responsibility of the authors and does not necessarily represent the official views of the National Institutes of Health.

## Conflict of interest

XW, MH, LF, OT, NG, and PK are employees of the Renal Research Institute, a wholly owned subsidiary of Fresenius Medical Care. PK holds stock in Fresenius Medical Care and receives author royalties from UpToDate and HS Talks.

The remaining authors declare that the research was conducted in the absence of any commercial or financial relationships that could be construed as a potential conflict of interest.

## Publisher’s note

All claims expressed in this article are solely those of the authors and do not necessarily represent those of their affiliated organizations, or those of the publisher, the editors and the reviewers. Any product that may be evaluated in this article, or claim that may be made by its manufacturer, is not guaranteed or endorsed by the publisher.
